# H-SLAM: Rao-Blackwellized Particle Filter SLAM Using Hilbert Maps

**DOI:** 10.3390/s18051386

**Published:** 2018-05-01

**Authors:** Guillem Vallicrosa, Pere Ridao

**Affiliations:** Underwater Robotics Research Center (CIRS), Computer Vision and Robotics Institute (VICOROB), Universitat de Girona, 17004 Girona, Spain; pere@eia.udg.edu

**Keywords:** AUV (Autonomous Underwater Vehicle), SLAM (Simultaneous Localization and Mapping), PF (Particle Filter), 2D

## Abstract

Occupancy Grid maps provide a probabilistic representation of space which is important for a variety of robotic applications like path planning and autonomous manipulation. In this paper, a SLAM (Simultaneous Localization and Mapping) framework capable of obtaining this representation online is presented. The H-SLAM (Hilbert Maps SLAM) is based on Hilbert Map representation and uses a Particle Filter to represent the robot state. Hilbert Maps offer a continuous probabilistic representation with a small memory footprint. We present a series of experimental results carried both in simulation and with real AUVs (Autonomous Underwater Vehicles). These results demonstrate that our approach is able to represent the environment more consistently while capable of running online.

## 1. Introduction

Robot localization is a fundamental problem in achieving true autonomy. Especially underwater, where global localization systems like Global Positioning System (GPS) are not available, vehicles have often to rely on Dead Reckoning (DR) navigation that drifts over time. This accumulated drift is problematic when constructing maps because a same geophysical feature may appear as a different one when it is re-observed after drifting.

To overcome this drift, systems like the Long Baseline (LBL), the Short Baseline (SBL), the Ultra-Short Baseline (USBL), the GPS Intelligent Buoyss (GIBs), or the single beacon navigation, are commonly used to provide absolute positioning fixes [1,2,3,4]. However, these systems require time for deployment and constrain the vehicle to their coverage area.

To avoid the use of external structures, a vehicle equipped with exteroceptive sensors such as sonars can make use of Terrain-Based Navigation (TBN) [5] to bound its navigational drift. However, detailed digital terrain maps are not always available. Moreover, those maps are mainly measured from surface ships, thus degrading their resolution as depth increases.

Another solution, is the use of Simultaneous Localization and Mapping (SLAM) methods [6,7], which do not require any external structures and neither a pre-obtained digital map. As in TBN, SLAM needs the use of exteroceptive sensors, mainly cameras or sonars. Although underwater cameras suffer from low visibility in turbid waters, they provide higher resolution and faster refresh rate while they are much cheaper than sonars. On the other hand, sonar sensors have lower resolution and refresh rate, but measure up to hundreds of meters regardless of water visibility issues.

Some of the most successful SLAM methods in the literature use a feature-based approach for SLAM [8,9,10]. Uniquely identifiable features are detected and associated to continuously correct the navigational drift and the learned map. However, underwater environments make robust feature extraction difficult, especially on sonar measurements, and a featureless method should be used. Featureless methods can rely on scan-matching, frequency registration, ⋯, where relations between different scans are obtained. Those relations are represented in a graph-like structure that can be solved/optimized with any of the state of the art back ends [11,12,13]. Another method is to rely on Particle Filters (PFs) where each particle carries its own map and is weighted against it for self-consistency of its measurements [14].

### 1.1. Underwater SLAM State of the Art

Focusing specifically on the underwater environment, multiple works have achieved successful SLAM implementations, either with optical imaging sensors or acoustic sonar sensors.

Optical imagery has been used to construct two-dimensional (2D) underwater photomosaics that correct the inherent DR drift and enable an overview of extended areas of the seafloor [15,16,17,18,19,20,21,22]. Additionally, in scenarios with a high three-dimensional (3D) component, optical imagery has also been used for 3D reconstructions [23,24,25,26,27]

Regarding sonar sensors, the Forward-Looking Sonar (FLS) provide a strong alternative to optical imagery mosaicking in low visibility conditions [28,29,30,31]. Although FLS provide a longer measurement range, its Field of View (FOV) is limited and the change of orientation greatly affects the perceived appearance of measured objects.

Multibeam echosounders are commonly used to obtain 2.5D elevation maps of the seafloor thanks to their wide swath and long range of measurements. Typically used on surface ships to map the seafloor, they are also used in Autonomous Underwater Vehicles (AUVs) to obtain a better resolution closer to the bottom [32,33,34,35].

Finally, mechanical scanning sonars and single beam echosounders have also been used for SLAM in man-made environments with line features [36]. Even in fully 3D environments like caves, with occupancy grids [37], as well as with scan-matching algorithms [38].

SLAM underwater is usually computed after the AUV is recovered from water and its data downloaded. After observing the obtained result, another mission can be scheduled to explore potential targets or cover the gaps of the first mission. This process can be inefficient and costly. However having the SLAM solution online, could enable autonomous exploration [39] or autonomous intervention [40] capabilities for the AUVs.

To the best of the authors knowledge the only underwater SLAM algorithms that have been tested online are [31,37]. The first uses multiple single beam echosounders and provides an Occupancy Grid (OG) map using an efficient Deferred-Reference Octree representation to avoid huge copies in its PF. While the second one uses a FLS Fourier-based registration with a pose-graph representation with loop-closing detection. While FLS mosaicking does not provide a useful representation of the environment for path planning, the OG grid map provides the perfect candidate for online path planning. OG describe the environment as free, occupied and unknown zones with certain probability. This information can be used to plan safe paths and autonomous exploration.

In our proposal, we want to work with occupancy maps because in future work they can be used for online path planning. To work with occupancy maps, we need to work with particle filters, where each particle carries their own version of the map. In [37] they reduced the memory footprint from OG maps by using an octree structure, but increased the computational complexity of the cell-query/update operation from constant O(1) to logarithmic O(log(n)). We propose a new SLAM framework, named Hilbert Maps SLAM (H-SLAM) which reduces the memory footprint of traditional OG maps while keeping the computational complexity constant O(1). Moreover, they offer a continuous occupancy representation that can be queried at any resolution.

### 1.2. Contribution

The main contributions of this paper are:

1. Bring the map representation named Hilbert Maps (HMs) to the underwater environment.
2. Implement a new SLAM framework, the H-SLAM.
  (a) Use sonar measurements with HM representation.
  (b) PF based.
  (c) Capable of running online on an AUV.
3. Simulated experiments and results of the method proposed.
  (a) Experiment with a known map. Localization only (TBN).
  (b) Full SLAM experiment.
4. Real experiments and results of the method proposed.
  (a) Datasets obtained by an AUV.

### 1.3. Paper Organization

The paper is organized as follows. Section 2 describes the HM representation and the specifics on how to use it for map localization. Section 3 presents the Rao-Blackwellized Particle Filter (RBPF) used in conjunction with the HM representation for the H-SLAM framework. Section 4 describes the datasets used for testing the algorithms while Section 5 discusses the results obtained with them. Finally, in Section 6, we present the conclusions.

## 2. Hilbert Maps

HMs where recently introduced in [41] to offer a continuous probabilistic representation of the space given a collection of range sensor measurements. In other words, it offers a continuous occupancy map representation. Unlike traditional OGs, there is no cell resolution, so any point in the space can be queried. Moreover, it captures spatial relationships between measurements, thus being more robust to outliers and possessing better generalization performance and exploiting that environments have some inherent structure. For example, if two close points are observed occupied the space between them will have a higher probability of being occupied than free while no other measurements are obtained on the neighbourhood.

Developed as an alternative to the Gaussian Process Occupancy Maps (GPOMs) [42], they offer similar advantages at a smaller computational cost. While GPOMs have a cubic computational cost $O(n^{3})$, HMs computational cost is constant O(1). Instead of training the classifier directly on the training points x, HMs project them to a finite set of features or inducing points Φ(x), where a simple logistic regression classifier is learned. Those features dot product approximates popular kernels in the Gaussian Process (GP) framework $k\left(x,x^{\prime }\right)\approx \Phi {\left(x\right)}^{T}\Phi \left(x^{\prime }\right)$, like the Radial-Basis Function. Furthermore, the logistic regression can be trained and updated using Stochastic Gradient Descent (SGD), making computation theoretically independent from the number of observations.

Given a dataset $D=\{x_{i},y_{i}\}$ where $x_{i}\in R^{D}$ is a point in the 2D or 3D space and $y_{i}\in \left\{-1,1\right\}$ is the label corresponding to the occupancy of the point $x_{i}$. HMs learn the discriminative model p(y|x,w) on the dataset through SGD. Once the model is learned, one can use the parameters w to predict the probability of occupancy of any query point $x_{*}$ as

(1) $$p\left(y_{*}=1|x_{*},w\right)=\frac{1}{1+\exp(-w^{T}\Phi \left(x_{*}\right))}\in \left[0,1\right].$$

**Figure 1:**
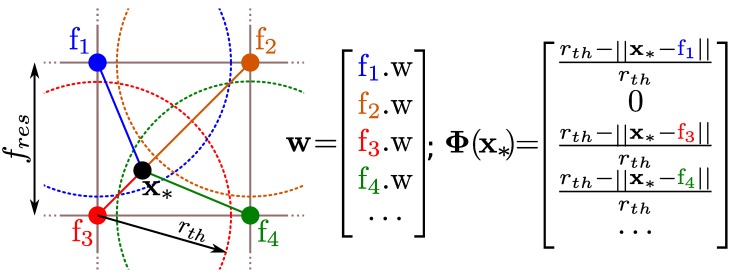
Schematic of a Hilbert Map. Features $f_{i}$ are spread at $f_{res}$ distance in a square grid and the neighbourhood that they affect is defined by the radius $r_{th}$. When predicting the occupancy of a point $x_{*}$, one must gather all the feature weights and multiply it by the value of the feature in that point $\Phi (x_{*})$ according to (1). In the example shown, the query point is outside $f_{2}$ neighbourhood and thus, its contribution is zero.

The most important parameters that define a HM are the learning rate of the SGD and features used. Regarding the learning rate $\eta_{t}$, it can be constant or decaying with time. Regarding the features, many different features have been applied to HMs [41,43,44], and the basic parameters common to them are the *feature_resolution* $f_{res}$, that defines how distant each feature are from each other, and the *radius_neighbourhood* $r_{th}$ that defines how far a feature affects its surroundings (Figure 1). The closer the features are, the smaller the details that can be represented. The lower the radius, the less features affect the same point in space. The feature used in this work is a simple triangle feature defined as

(2) $$\Phi \left(x\right)=\left\{\begin{array}{cc} \frac{r_{th}-r}{r_{th}} & \;\mathrm{if}\;r<r_{th}\\ 0 & \;\mathrm{otherwise}\; \end{array}\right.$$

where $r=\left|\right|f_{i}-x{\left|\right|}_{2}$ and $f_{i}$ is the position of the feature *i*.

**Figure 2:**
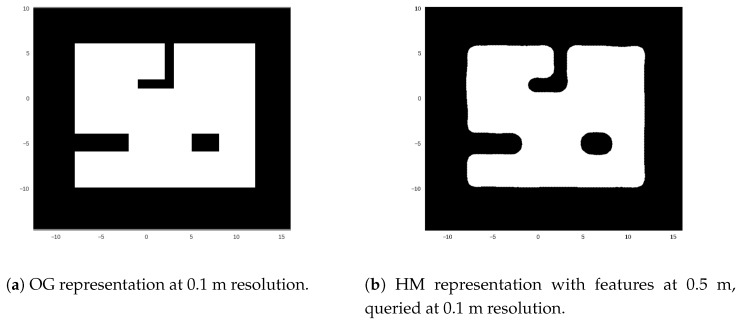
Comparison between OG and HM representation queried at same resolution. Notice that rounded corners are not the most desirable representation for structured environments, but for underwater scenarios is not usually a drawback.

Being a continuous representation features can be much farther than cells in a traditional OG, but achieve a similar representation at a much lower memory footprint. For example the map described in Figure 2 extends 28.5 × 24.5 m which for an occupancy grid at 0.1 m resolution takes around 70,000 cells to represent. If represented by doubles (8 bytes/double), it takes ≈545.5 kB. However a HM representation at 0.5 m feature resolution, takes ≈21.8 kB (a 0.04% of the memory) providing similar representation at 0.1 m queries.

### Hilbert Map Learning and Raycasting

**Figure 3:**
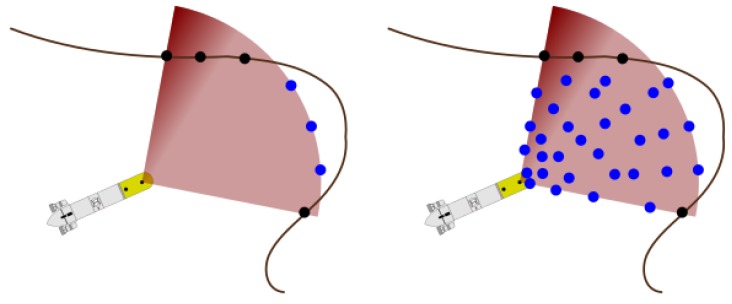
(**left**) Original range measurements made with a sonar. (**right**) Sampled points for map learning (black points are occupied and blue points are free).

Learning a map from range sensors measurements and querying a point in the map, are both clearly defined in the seminal work of HMs [41]. To include range measurements, they are first discretized into single points. The point at the end of the range is labeled free if the range is maximum and occupied otherwise. Then, the rest of the ray (from vehicle position to measured range) is sampled randomly and labeled free every 1 or 2 m to properly cover the ray (Figure 3). Those points and labels are learned into the HM.

However, to develop a SLAM framework based on HMs, it lacks a necessary raycast method to compare the real range measurements with the expected range measurements that the vehicle would have according to the learned map. On grided OG maps, the cells are queried through the ray path until an occupancy value bigger than a threshold is found [45]. Our HM raycasting method is inspired by the one developed on GPOMs [46].

**Figure 4:**
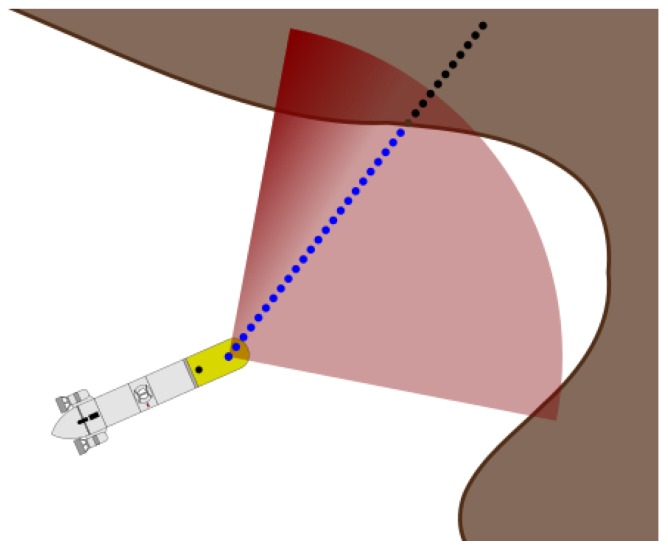
Example of raycast where queries are made at specific resolution.

The raycast starts from the vehicle position in the HM and points in the same relative direction as the real measurement. Points at increasing distance from the vehicle are queried in the HM to obtain the occupancy value (Figure 4). This distance is defined as the query resolution. When a query point has an occupancy value bigger than a threshold, this point is considered a hit (occupied) and no more points are queried. To get the exact position where the threshold was crossed, a linear interpolation between the hit point and the point previous to the hit point is computed. Finally, the raycasted range is the distance between the vehicle position and the result of the linear interpolation.

## 3. Rao-Blackwellized Particle Filter with Hilbert Maps

AUVs are often loaded with a handful of sensors to provide proper positioning. Depth sensor, Attitude and Heading Reference System (AHRS) and Doppler Velocity Log (DVL) provide excellent positioning except for the *x* and *y* axis in the absence of GPS, SBL, LBL, USBL or GIB. To assess this positioning one can represent the state of the vehicle as a RBPF [47]. Here, states directly observable using vehicle sensors are removed from the PF and are tracked by a single Extended Kalman Filter (EKF) shared by all particles whose state vector is

(3) $$x_{k}^{ekf}={\left[z_{k}\;\;u_{k}\;\;v_{k}\;\;w_{k}\right]}^{T},$$

where $z_{k}k$ is the depth of the vehicle in the world frame, $\left[u_{k}\;\;v_{k}\;\;w_{k}\right]$ are the velocities in the vehicle frame at the time *k*. The vehicle orientation $\phi_{k},\theta_{k}$ roll and pitch and the yaw rate ${\dot{\psi }}_{k}$ in the world frame are taken as inputs $u_{k}$ of the EKF prediction model and are not estimated. The remaining states are estimated by the PF, where each particle is defined as

(4) $$x_{k}^{pf,i}=\left\{{\left[x_{k}^{i}\;\;y_{k}^{i}\;\;\psi_{k}^{i}\right]}^{T},\;w_{k}^{i},\;m_{k}^{i}\right\},$$

where *i* is the particle index and ${\left[x_{k}^{i}\;\;y_{k}^{i}\;\;\psi_{k}^{i}\right]}^{T}$ are the positions and the yaw in the world frame, $w_{k}^{i}$ is the weight of the particle and $m_{k}^{i}$ is the HM of the particle.

The particle filter is initialized from the on-board DR filter if an absolute positioning system is available. Otherwise the filter is initialized at the origin for x,y and uses the current sensor measurements to initialize the state model.

### 3.1. State Propagation

At each sensor measurement, the EKF is predicted to the time of the observation. A simple constant velocity model is used for the prediction as

(5) $$x_{k+1}^{ekf}=f\left(x_{k}^{ekf},u_{k},n_{k}\right)=\left[\begin{array}{c} z_{k}+\cos\left(\theta_{k}\right)\cos\left(\phi_{k}\right)\left(w_{k}t+n_{w_{k}}\frac{t^{2}}{2}\right)\\ u_{k}+n_{u_{k}}t\\ v_{k}+n_{v_{k}}t\\ w_{k}+n_{w_{k}}t \end{array}\right]$$

where *t* is the time increment from the previous prediction, $u_{k}={\left[\phi_{k}\;\;\theta_{k}\;\;{\dot{\psi }}_{k}\right]}^{T}$ is the input control vector and $n_{k}={\left[n_{u_{k}}\;\;n_{v_{k}}\;\;n_{w_{k}}\right]}^{T}$ are the acceleration noises in the linear velocities. Note that noises in roll and pitch $\left[n_{\phi_{k}}\;\;n_{\theta_{k}}\right]$ are so small that can be considered negligible and are not taken into account. Covariance is also predicted as

(6) $$P_{k+1}=F_{k}P_{k}F_{k}^{T}+W_{k}Q_{k}W_{k}^{T}$$

where $F_{k}={\left.\frac{\partial f(x_{k}^{ekf},u_{k},n_{k})}{\partial x_{k}^{ekf}}\right|}_{x_{k}^{ekf}={\hat{x}}_{k}^{ekf},n_{k}=0}$, $W_{k}={\left.\frac{\partial f(x_{k}^{ekf},u_{k},n_{k})}{\partial n_{k}}\right|}_{x_{k}^{ekf}={\hat{x}}_{k}^{ekf},n_{k}=0}$, and $Q_{k}=diag\left\{\sigma_{u}\;\;\sigma_{v}\;\;\sigma_{w}\right\}$.

Each particle is also predicted forward by randomly sampling the uncertainties of $u_{k},v_{k}$ from the EKF and a user specified yaw rate uncertainty $\sigma_{\dot{\psi }}$. The velocities and their covariances are transformed for each particle from the body frame to the world frame {W} as

(7) $$\begin{array}{c} \left[\begin{array}{c} {}^{W}{\dot{x}}_{k}^{i}\\ {}^{W}{\dot{y}}_{k}^{i}\\ {}^{W}{\dot{z}}_{k}^{i} \end{array}\right]=Rot\left(\phi_{k},\theta_{k},\psi_{k}^{i}\right)\left[\begin{array}{c} u_{k}\\ v_{k}\\ w_{k} \end{array}\right] \end{array}$$

(8) $$\begin{array}{c} {}^{W}P_{{\dot{x}}_{k},{\dot{y}}_{k},{\dot{z}}_{k}}^{i}=Rot\left(\phi_{k},\theta_{k},\psi_{k}^{i}\right)P_{u_{k},v_{k},w_{k}}Rot{\left(\phi_{k},\theta_{k},\psi_{k}^{i}\right)}^{T} \end{array}$$

where $Rot(\phi_{k},\theta_{k},\psi_{k}^{i})$ is a rotation matrix given the attitude Euler angles and $P_{u_{k},v_{k},w_{k}}$ is the 3×3 sub-matrix of $P_{k}$ containing the velocity uncertainties. Those obtained values are used to predict each particle positions as

(9) $$\begin{array}{c} x_{k+1}^{i}=x_{k}^{i}+N\left({}^{W}{\dot{x}}_{k}^{i},{}^{W}P_{{\dot{x}}_{k},{\dot{x}}_{k}}^{i}\right)t \end{array}$$

(10) $$\begin{array}{c} y_{k+1}^{i}=y_{k}^{i}+N\left({}^{W}{\dot{y}}_{k}^{i},{}^{W}P_{{\dot{y}}_{k},{\dot{y}}_{k}}^{i}\right)t \end{array}$$

(11) $$\begin{array}{c} \psi_{k+1}^{i}=\psi_{k}^{i}+N\left({\dot{\psi }}_{k},\sigma_{\dot{\psi }}\right)t \end{array}$$

where $\dot{\psi }$ is taken from $u_{k}$.

### 3.2. State Update

Once the prediction has been computed up to the time of the sensor measurement, the EKF state can be updated with the common EKF update equations. The measurement function is defined as

(12) $$z_{k}=H_{k}x_{k}^{ekf}+v_{k}$$

where $z_{k}$ is the measurement, $H_{k}$ defines which states are observed and $v_{k}$ is the noise of the measurement.

Depending on the different measurements $z_{k}$ provided by the different sensors (see Section 4.2) the $H_{k}$ matrix will change. For example, the depth sensor provides depth measures and it is defined as

(13) $${\left[\begin{array}{c} z \end{array}\right]}_{depth}=\left[\begin{array}{cccc} 1 & 0 & 0 & 0 \end{array}\right]x_{k}^{ekf}+\left[\begin{array}{c} \sigma_{depth} \end{array}\right]$$

DVL sensor provides velocities in the vehicle frame, and thus it is defined as

(14) $${\left[\begin{array}{c} u\\ v\\ w \end{array}\right]}_{DVL}=\left[\begin{array}{cccc} 0 & 1 & 0 & 0\\ 0 & 0 & 1 & 0\\ 0 & 0 & 0 & 1 \end{array}\right]x_{k}^{ekf}+\left[\begin{array}{c} \sigma_{u}\\ \sigma_{v}\\ \sigma_{w} \end{array}\right]$$

Finally, AHRS sensor provides orientation in roll and pitch, and angular rate in yaw $\left[\phi \;\;\theta \;\;\dot{\psi }\right]$ that are saved in the input control vector $u_{k}$.

### 3.3. Weighting, Learning and Resampling

Once a sonar measurement is received, it is segmented according to the returned intensities to obtain a single range and occupancy value. If no significant intensity is found, the range is set to the maximum range value and the measure is set to *free*. Otherwise, the range is set to the range of the highest intensity and the measure is set to *occupied*.

If it is an *occupied* measurement, its range $r_{k}^{meas}$ is compared with each particle map $m_{k}^{i}$ to update the particle weight. The expected range measurement $r_{k}^{i,cast}$ is obtained by casting a ray as described in Section 2.1, from the particle position in their respective HM $m_{k}^{i}$. The weight update per each particle is proportional to the difference of those ranges

(15) $$w_{k+1}^{i}\propto w_{k}^{i}\exp\left(-\frac{{\left(r_{k}^{meas}-r_{k}^{i,cast}\right)}^{2}}{\sigma_{r}^{2}}\right),$$

where $\sigma_{r}$ is the range measurement covariance. This can be thought as a measure of self-consistency of the each particle HM.

After particle weighting, the measurement is learned in each $m_{k}^{i}$ to be used in future weightings and to properly reconstruct the environment. These ranges are first sampled and then learned as points as explained in Section 2.1.

Finally, the well known Sequential Importance Resampling (SIR) is used each time the number of effective particle $N_{eff}$ falls below half of the number of particles ($N_{eff}<N/2$) [48].

Please note that in the case of TBN, particles carry no HMs and there is a single shared HM. This shared map is only learned beforehand and never updated. The learning step is suppressed in this case.

## 4. Datasets

The proposed H-SLAM framework was tested on several datasets. First on a synthetic dataset to ensure correct implementation and to be able to compare against ground truth, and then with two underwater datasets, one structured and one non-structured, gathered by an AUV.

### 4.1. Simulated Dataset

**Figure 5:**
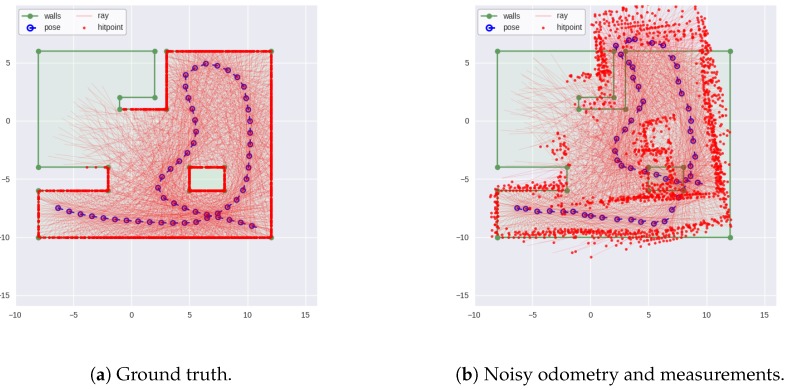
Simulated dataset of an indoor environment. Vehicle starting position on the bottom left.

This dataset is used as a proof-of-concept of the algorithms. The dataset is generated from a set of 53 vehicle poses in a 2D map where 36 range measurements spaced $10^{\circ }$ around the vehicle are obtained for each pose (Figure 5a). The increments between the poses are obtained, then linear and angular gaussian noises are added to obtain the odometry measurements. The range measurements are also corrupted by gaussian noise (Figure 5b). When predicting particles, odometry increments [ΔxΔyΔψ] are combined with gaussian noise $\left[\sigma_{lin}\;\;\sigma_{lin}\;\;\sigma_{ang}\right]$ to obtain particle positions.

**Figure 6:**
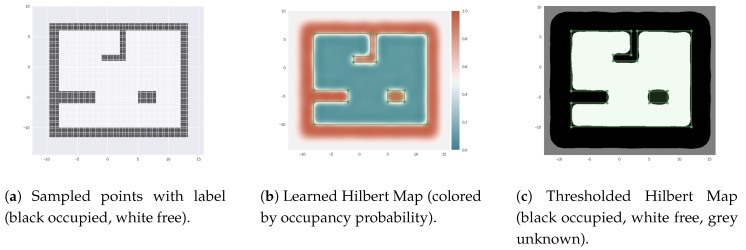
(**a**) Sampled points from the simulated scenario; (**b**) Learned HM for TBN; (**c**) Learned HM thresholded at p(occ)>0.5 for the occupied, p(occ)<0.5 for free, and p(occ)=0.5 for unknown, to better identify the different areas.

This dataset is used for both TBN and SLAM. For the TBN case, the original map is sampled at 0.2 m resolution and those points are used to learn its HM representation (Figure 6). Then this map is used to localize the particles. On the SLAM case, only the noisy odometries and ranges are used as input to the filter because each particle learns its own HM $m_{k}^{i}$.

Using only odometry increments and ranges simplifies the filter explained in Section 3. Each particle state is propagated by compounding their current position with the noisy odometry increments.

### 4.2. Real-World Datasets

**Figure 7:**
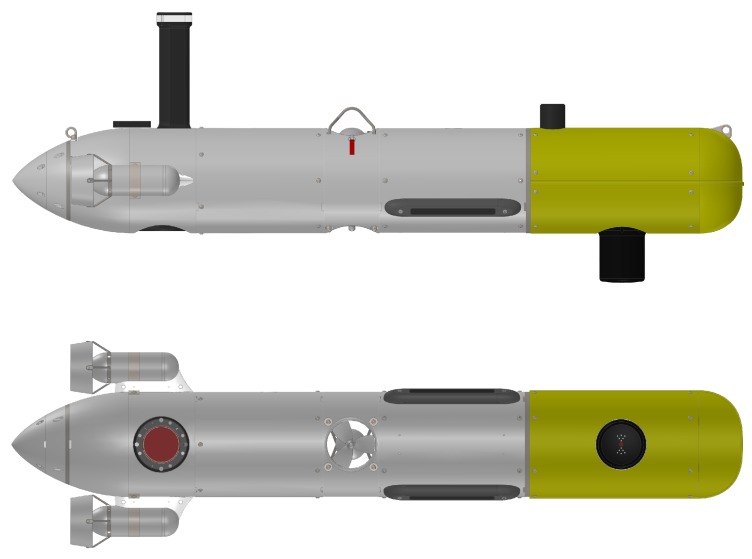
Sparus II AUV side view (**up**) and bottom view (**bottom**). Profiler is mounted on the payload area (in yellow) at the bottom of the vehicle.

These datasets were obtained with Sparus II AUV [49] equipped with a Tritech SeaKing Profiling Sonar for range measurements. The Sparus II AUV provides depth information from a pressure sensor, velocities and altitude from a DVL, and attitude from an AHRS. The profiler is mounted at the payload space of the AUV (Figure 7).

**Figure 8:**
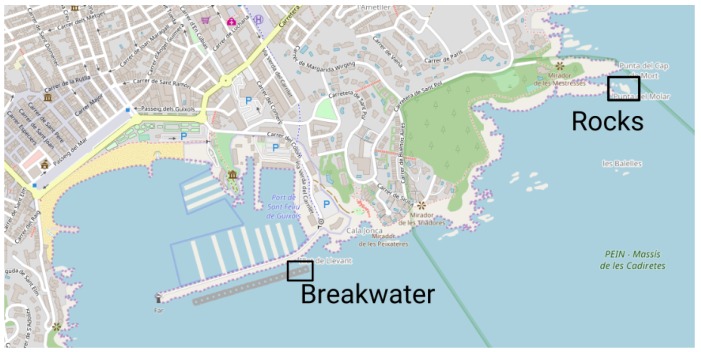
Location of both real-world datasets along Sant Feliu de Guixols’ coast (source: OpenStreetMap©).

With those sensors the AUV is capable to provide a DR navigation that drifts over time as can be observed in the following datasets. Both datasets were taken along Sant Feliu de Guixols’ coast (Figure 8) at a constant depth, during the experiments regarding [50] trials.

No GPS or USBL were available to provide global corrections to the navigation drift or to provide a ground truth to compare with. The profiler provides a $120^{\circ }$ FOV in the front of the vehicle at $1.8^{\circ }$ angular increments. This forward-looking configuration complicates the SLAM in the sense that until a loop is closed, same locations are not measured again.

Each ray measurement provides ranges from 0 m to 10 m at 0.025 m resolution with their corresponding intensity values. Those rays are thresholded according to a minimum and maximum range, and a minimum return intensity to obtain a range measurement to be used in the H-SLAM filter.

**Figure 9:**
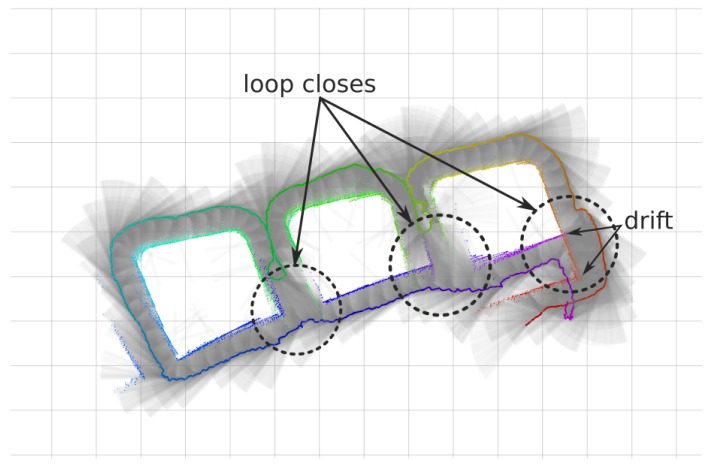
DR trajectory in a continuous line from magenta to red, with the corresponding profiler rays (in black) and their hitpoints (also colored by time) of the breakwater dataset. Grid cells at the background are 5 m wide.

The first dataset was taken on the man-made breakwater structure outside of the harbour. The three most eastern blocks of around 14 × 14 m with a spacing of 5 m were surveyed with the AUV (Figure 9). The dataset contains a total of 12,412 range measurements over 15 min mission at 1.5 m constant depth. As can clearly be observed on the figure, when the vehicle returns to the starting point the drift is clearly noticeable. This dataset contains three loop closes, where same features are re-observed after going around each of the three blocks. 

**Figure 10:**
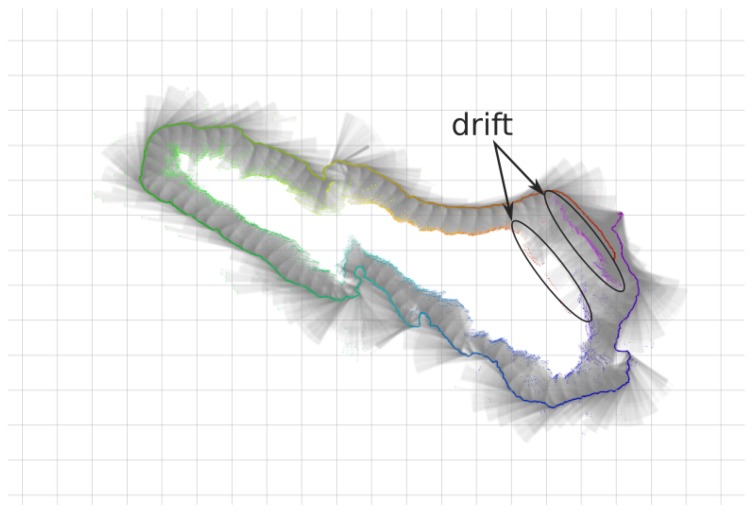
DR trajectory in a continuous line from magenta to red, with the corresponding profiler rays (in black) and their hitpoints (also colored by time) of the rocks dataset. Grid cells at the background are 5 m wide.

The second dataset was taken on the natural rock structure next to the so-called *Punta del Molar*. Like the previous dataset, the AUV navigated around the rock (Figure 10). The dataset contains a total of 14,417 range measurements over 17 min mission at 2.5 m constant depth. Likewise the first dataset, the drift is clearly observable when the vehicle returns to the starting position.

## 5. Results

All the tests on the different datasets were run with the similar parameters to ease the comparison of results (Table 1). Feature resolution and radius of the neighbourhood were increased for the real datasets since they are bigger than the simulated one and have less details. Range covariance was also increased due to the bigger errors obtained when dealing with real sensors.

### 5.1. Simulated Dataset

**Figure 11:**
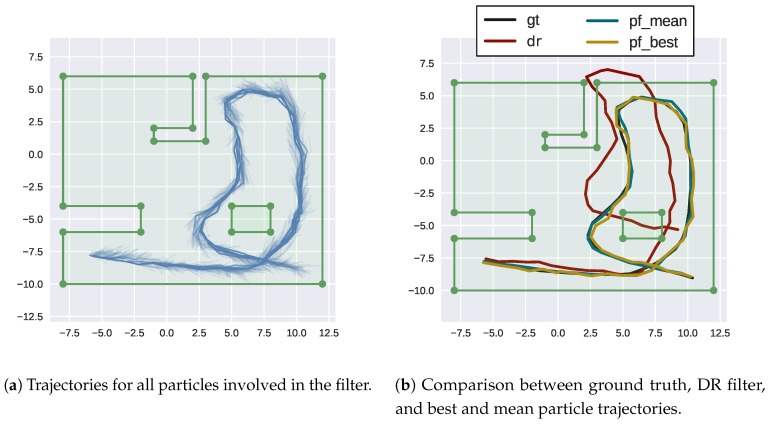
Trajectory results of TBN on HMs.

The simulated dataset is first used on a TBN experiment, where the HM is first learned from samples as explained on Section 4.1. This map is shared between particles being only queried to modify particle weights according to the differences between measured and casted rays. The results are compared against the ground truth but also against the provided odometry inputs in a DR filter that simply composes them (Figure 11).

**Figure 12:**
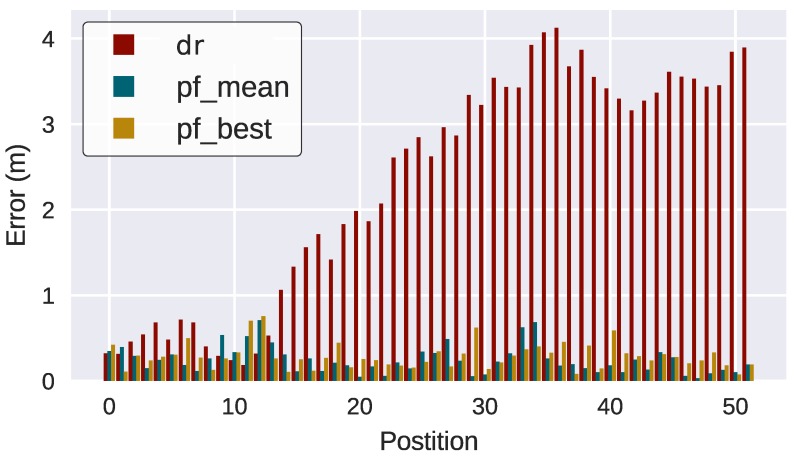
Position error of the filters compared to ground truth (TBN).

As can be observed, the TBN corrects the vehicle trajectory reducing significantly the position error. While the DR filter error keeps increasing, the TBN error is maintained almost constant around 0.4 m (Figure 12).

**Figure 13:**
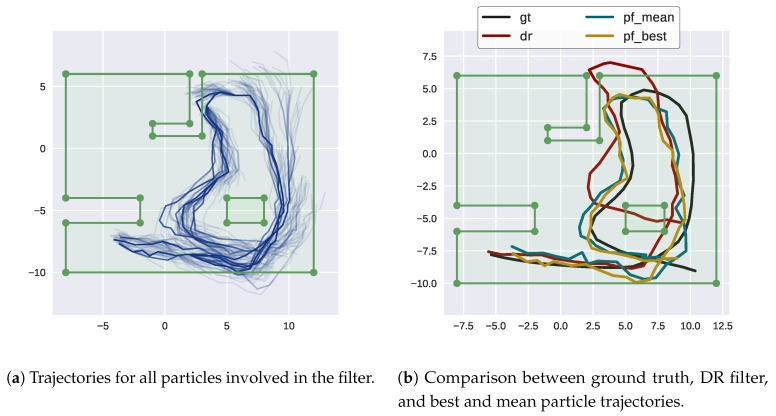
Trajectory results of SLAM on HMs.

As expected, moving to SLAM increases the error and the correction of the trajectory is lower than in the TBN case (Figure 13).

**Figure 14:**
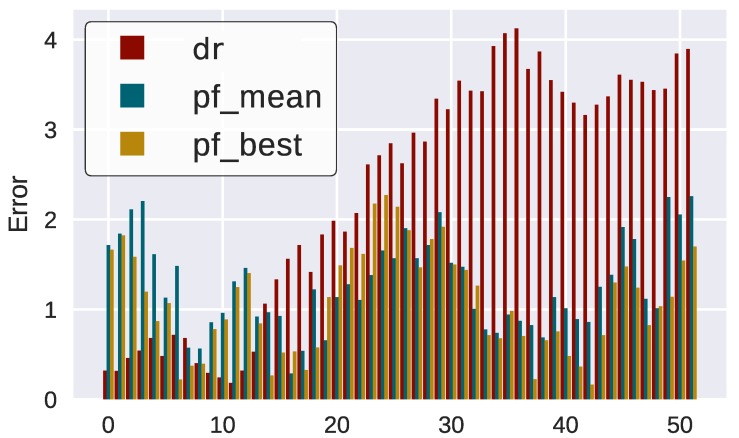
Position error of the filters compared to ground truth (SLAM).

However, errors continue to be bounded although they are much higher due to the nature of map incremental learning and self-consistency checks (Figure 14).

**Figure 15:**
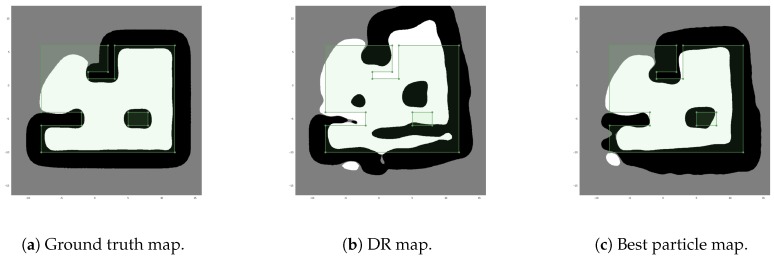
Comparison of HMs learned from different vehicle trajectories.

Another way to compare the results is to compare the map learned using ground truth odometry and measurements against the map learned by the DR filter and the H-SLAM filter (Figure 15). In this case, the representation obtained by the H-SLAM is much more close to the ground truth one than the one obtained by the DR filter.

### 5.2. Breakwater Dataset

On the case of the breakwater dataset, we can observe a quite problematic area in the small corridors between the blocks. A multipath echo is clearly present when looking to the east. This multipath returns a maximum range which is interpreted as a completely free ray. This problem is clearly visible on the rightmost block, causing the HM to represent it hollow.

**Figure 16:**
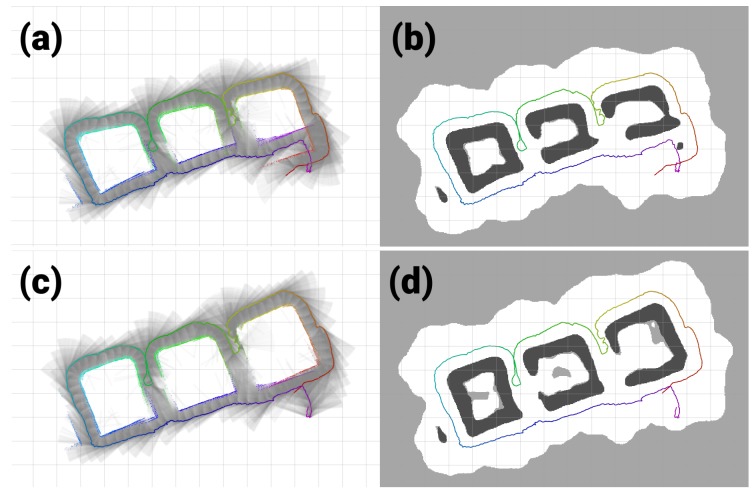
Breakwater dataset results. (**a**,**b**) Original dataset; (**c**,**d**) Corrected dataset; (**a**,**c**) Raw rays and endpoints with the vehicle trajectory colored by time; (**b**,**d**) Learned HM segmented to show free/unknown/occupied values with the vehicle trajectory.

When the H-SLAM is applied to the breakwater dataset, the result clearly improves over the trajectory, providing more consistent sizes for the blocks and avoiding the double wall at the end of the dataset (Figure 16). Observing the reprojected measurements on the corrected trajectory, no major drifts are observed.

**Figure 17:**
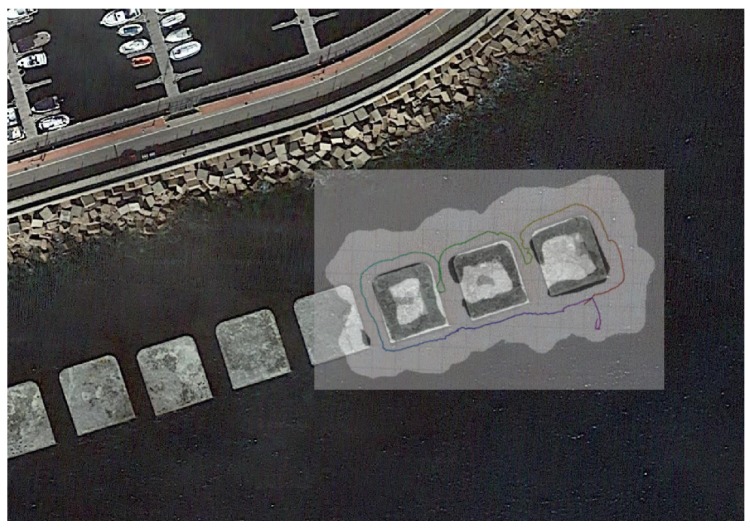
Breakwater HM superimposed with a satellite image (source: Map data ©2018 Google, Inst. Geogr. Nacional, Spain).

The validity of H-SLAM approach can be seen when comparing the results with the satellite images because they maintain the same structure as they have underwater (Figure 17).

**Figure 18:**
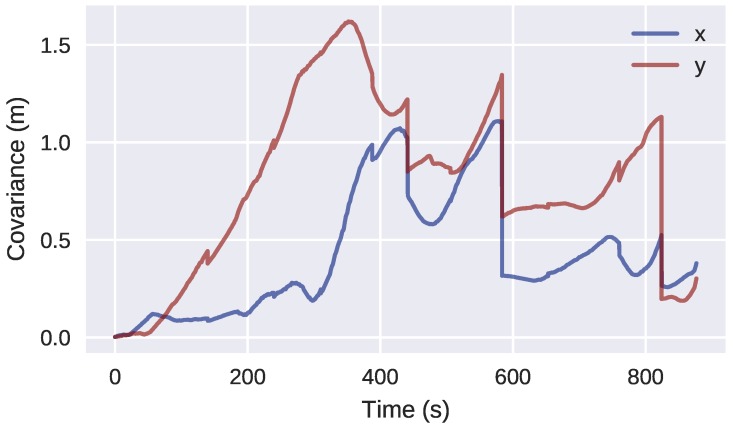
Covariance of the particles over time for the Breakwater dataset.

Finally, observing the covariance of the particles over time (Figure 18), the three loop closing events described in Section 4.2 produce a clear decrease in uncertainty of the H-SLAM localization.

### 5.3. Rocks Dataset

**Figure 19:**
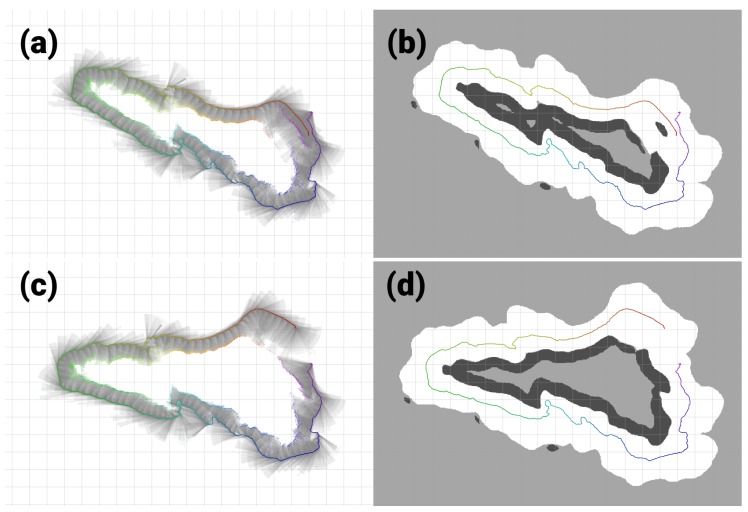
Rocks dataset results. (**a**,**b**) Original dataset. (**c**,**d**) Corrected dataset. (**a**,**c**) Raw rays and endpoints with the vehicle trajectory colored by time. (**b**,**d**) Learned HM segmented to show free/unknown/occupied values with the vehicle trajectory.

On the rocks dataset the same parts of the map are not observed until the trajectory finishes. Incremental corrections are made during the whole dataset and at the end a loop-closing is achieved. Several outliers are observed at the boundaries of the dataset due to proximity to other rock formations (Figure 19).

**Figure 20:**
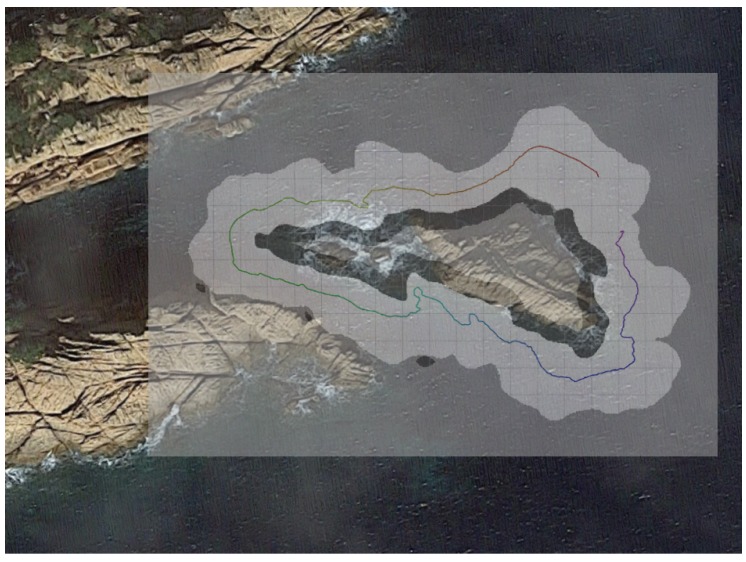
Rocks HM superimposed with a satellite image (source: Map data ©2018 Google, Inst. Geogr. Nacional, Spain).

Although the natural rock structure does not maintain the same structure underwater, when comparing the results with the satellite images, the validity of H-SLAM can be seen (Figure 20). Furthermore, the small occupied spots on the south-western part of the explored zone are clearly caused by the nearby rock structures.

**Figure 21:**
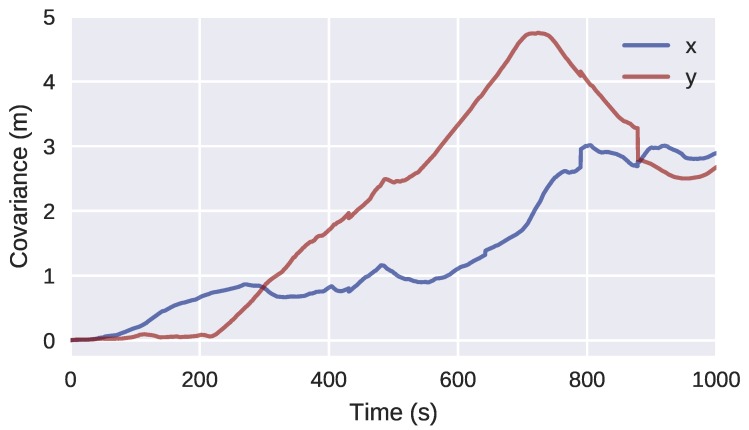
Covariance of the particles over time for the Rocks dataset.

Finally, observing the covariance of the particles over time (Figure 21), a loop closing event is observed at around 900 s that corresponds to revisiting the initial area.

### 5.4. Performance

H-SLAM was not run online when obtaining the datasets, but from previously obtained datasets saved in a *rosbag* file. This file, part of the Robot Operating System (ROS) [51], allows to replay data exactly as how it was obtained. In this case, the algorithm was run as fast as possible through the *bagfile* to compare the time it took to gather data (total available time for execution) against the time needed to compute the H-SLAM solution (Table 2).

As can be observed, the computing time is much lower an thus making the algorithm capable of running online on the AUV, even with many more particles than the 40 used on the tests.

## 6. Conclusions

In this work, we have presented new SLAM framework named H-SLAM for AUVs equipped with sonars. The combination of a RBPF with a HM representation of the environment provided trajectory corrections that increased the consistency of the recorded measurements both in simulation and in real datasets. Moreover, the computing time required is much lower than the time it took to collect the datasets, being capable of being used online on an AUV.

In the simulated datasets, the RBPF provided a significant correction when used for TBN with a known map, and a lesser correction when used for SLAM. However the final map was much more consistent than the one obtained by the DR filter.

In the real datasets, significantly more consistent maps were also obtained. Especially on the breakwater dataset, the multiple closing loops allowed to obtain a correct trajectory and map that matches the satellite image of the structure.

## 7. Future Work

The algorithms have been tested at constant depth providing continuous occupancy maps in 2D. Future work must better reflect the nature of underwater environments, extending H-SLAM to the 3D case. Moreover, multipath errors observed on the real datasets should be filtered out. Our idea is check the range measurements persistence over time before using them in H-SLAM.

## Figures and Tables

**Table 1:** Parameters used in the different datasets. On the real datasets prediction covariances are gathered from the covariance matrix $P_{k}^{ekf}$.

Parameter	Simulated	Breakwater	Rocks
Feature resolution (m)	0.5	1.0	1.0
Radius neighbourhood $r_{th}$ (m)	1.5	2.0	2.0
Linear covariance $\sigma_{lin}$ (m)	0.25	-	-
Angular covariance $\sigma_{ang}$ (degree)	2	-	-
Range covariance $\sigma_{r}$ (m)	0.05	0.4	0.4
Number of particles	40	40	40

**Table 2:** Computing time comparison with dataset collection time.

	Breakwater	Rocks
Time to obtain dataset	14 min 36 s	16 min 54 s
Time to run H-SLAM	02 min 26 s	03 min 42 s
